# Immunohistochemical Expression of the Alpha Nicotinic Acetylcholine Receptor 7 in the Human Normal, Diabetic, and Preeclamptic Placenta and Products of Conception

**DOI:** 10.3389/fphys.2020.607239

**Published:** 2020-11-25

**Authors:** Ahmad Alwazzan, Riffat Mehboob, Syed Amir Gilani, Amber Hassan, Shahida Perveen, Imrana Tanvir, Humaira Waseem, Kashifa Ehsan, Fridoon Jawad Ahmad, Javed Akram

**Affiliations:** ^1^Division of Gynecology Oncology, Faculty of Medicine, King Abdulaziz University,, Jeddah, Saudi Arabia; ^2^Research Unit, Faculty of Allied Health Sciences, The University of Lahore, Lahore, Pakistan; ^3^SISSA, International School for Advanced Studies, Trieste, Italy; ^4^Department of Pathology, Continental Medical College Lahore, Lahore, Pakistan; ^5^Department of Pathology, King Abdulaziz University, Rabigh, Saudi Arabia; ^6^Shukat Khanum Memorial Hospital, Lahore, Pakistan; ^7^Physiology and Cell Biology Department, University of Health Sciences Lahore, Lahore, Pakistan; ^8^Institute of Regenerative Medicine, University of Health Sciences Lahore, Lahore, Pakistan

**Keywords:** placenta, products of conception, nicotine receptors, alpha 7 acetylcholine receptor, sudden perinatal deaths

## Abstract

Preeclampsia (PE) and gestational diabetes (GD) are complications in advanced pregnancy while miscarriage for early pregnancy. However, the etiological factors are not well understood. Smoking has been associated with these complications as well as the sudden intrauterine deaths, sudden infant death, miscarriages, and still births. However, the immunolocalization of alpha 7 nicotine acetylcholine receptor (α7-nAChR) is not studied.

**Materials and Methods:** α7-nAChR subunit expression was evaluated in 10 paraffin-embedded placental tissues after delivery and 10 tissue samples of products of conception during first trimester by immunohistochemistry. Among the placental tissues, two samples were normal placental tissue, four from PE mother, and four from GD mother. The expression of α7-nAChR was compared between the two groups in general and within the subgroups of placenta as well. Protein expression was evaluated using the nuclear labeling index (%) of villi with positive cells stained, positive cells in the decidua, and intensity of staining in the outer villous trophoblast layer.

**Results:** The expression of α7-nAChR protein was high in all the cases of placenta and products of conception (POCs). α7-nAChR expression showed no notable differences among different cases of miscarriages irrespective of the mother’s age and gestational age at which the event occurred. However, there were some changes among the normal, PE, and GD placental groups in the linings of the blood vessels. Changes were restricted to the villi (as opposed to the decidua) lining cells, both cytotrophoblast and syncytiotrophoblast, and were specific to the α7 subunit. PE blood vessel lining was thicker and showed more expression of this receptor in endothelial cells and myofibroblasts in PE and GD groups. In POCs, the strong expression was observed in the decidua myocytes of maternal blood vessels and in syncytiotrophoblast and cytotrophoblast of chronic villi.

**Conclusion:** Nicotine acetyl choline receptors are found to be expressed highly in the placental tissues and in products of conception. They may be associated with the sudden perinatal deaths and miscarriages or complications of pregnancy.

## Introduction

The prevalence of preeclampsia (PE) and gestational diabetes (GD) during pregnancy is 2–8% (Umesawa and Kobashi, 2017), respectively. The hazardous effects of exposure to tobacco smoking, whether active or passive, during pregnancy include placenta previa (Shobeiri and Jenabi, 2017), placental abruption (Ananth et al., 2016), ectopic pregnancy, early membrane rupture, low birth weight (Bar-Zeev and Solt, 2018), premature birth, sudden perinatal deaths, and stillbirths (Lavezzi et al., 2017; Anderson et al., 2019). We have highlighted these aspects in previous studies as well (Lavezzi et al., 2010, 2011, 2020; Mehboob, 2017; Mehboob et al., 2017; Muhammad et al., 2018).

Tobacco smoke contains many neurotoxic ingredients, but nicotine is the one with highest adverse effects on neurotransmission such as cholinergic system during the development of central nervous system in fetus (Klenowski and Tapper, 2018). Acetylcholine (ACh) is the main cholinergic neurotransmitter and has a basic functional role in the development of nervous system via its synaptic mechanisms of its nicotinic Ach receptors (nAChs) (Brown, 2019). There are nine nAChR subunits: α2, α3, α4, α5, α7, α9, β1, β2, and δ. Nicotine can mimic the effect of ACh and incorrectly signal the cholinergic system to activate when there is no requirement (Robbins, 2016).

Particularly, the α7 subunit affects the developing nervous system in a toxic way and damages the neuronal differentiation, angiogenesis, axon formation, and synaptic transmission (Cross et al., 2017; Lavezzi, 2018). This phenomenon highlights the vulnerability of α7-nAChRs as potential targets for other neurotoxicants, apart from nicotine, during the critical developmental phases of brain. A study was conducted to test this hypothesis, and it was observed that chlorpyrifos, an organophosphate pesticide used in agriculture, exhibits similar actions to those of nicotine in stimulating α7-nAChRs (del Pino et al., 2016). Previous studies have indicated that α2– 7-, 9, 10-, and β1-nAChR subunits are localized in the placenta (Ghazavi et al., 2013; Machaalani et al., 2014, 2018)) but with varying expressions depending on the structure and type of cell. However, a study that is detailed and in different populations has not been conducted yet.

Hence, the current study aimed to evaluate the immunohistochemical expression and localization of α7-nAChRs in three groups of placenta (normal, preeclamptic, and gestational diabetic) after delivery and retained products of conception (POCs) after miscarriage in the first trimester. The purpose is to assess the association with miscarriages and sudden perinatal deaths and explore the involvement of pesticides and smoking exposure in developing countries like Pakistan.

## Materials and Methods

### Tissue Collection

Ten placental tissue samples, comprising of two normal, four PE, and four GD samples, were obtained from the Obstetrics and Gynecology Department, after the ethical approval of The University of Lahore Teaching Hospital, Lahore, Pakistan. Placentas were collected within the first hour of delivery. Additionally, the retained POC tissue samples were collected from patients having miscarriages during the first trimester. Four small (2 × 2 cm) separate samples were obtained systematically from different areas of the POCs and placentas. Placental samples were obtained from the maternal side, code side alveoli, fetal side, and necrotic area and allowed to get fixed in 10% formalin for 24 h. Samples were embedded in paraffin and stored at room temperature for sectioning. Sections of 5 μm were cut using a microtome and mounted on 3-aminopropyltriethoxysilane (APES)-coated slides in preparation for staining. Tonsil tissue was used as a positive control for α7-nAChR receptor.

### α7-nAChR Immunohistochemistry

Paraffin-embedded sections were deparaffinized with different grades of ethanol (100–70%), two washes in xylene for 5 min each and three washes in phosphate-buffered saline (PBS) (pH 7.4). Antigen retrieval was carried out by citrate buffer, heated for 60°C. Hydrogen peroxidase was used for blocking the endogenous peroxidase activity for 30 min at 4°C. Non-specificity in the tissue section was blocked by 10% normal rabbit serum and then incubated with the rabbit polyclonal, α7-nAChR primary antibody (Abcam, ab10096, 1:200 dilution) at 4°C overnight. Tissue sections were washed in PBS and incubated with goat antirabbit immunoglobulin G (IgG) secondary antibody (PK-6101, Vector Laboratories, CA, United States). Sections were further processed with the avidin–biotin immunoperoxidase technique (VEDH-4000, Vector Laboratories, CA, United States), counterstained with 3,3′-diaminobenzidine (DAB) and coverslipped.

### Statistical Analysis

Data were analyzed by using SPSS 25.0. All the quantitative variables were presented in mean ± SD. Independent sample t test was applied to check the mean difference in both groups (placenta and POCs). *P* < 0.05 was considered as significant.

### α 7-nAChR Expression Quantification

Immunoreactivity in the tissue sections was assessed in each randomly selected nucleus and cell as the number of cells exhibiting a dark brown stain, divided by the total cells, and shown as% [nAChR immunopositivity index (nAChR-I)] (Table 1) (Lavezzi et al., 2014).

**TABLE 1:** Scoring scale for quantification of staining.

0	0–1%	Negativity
+ 1	Less than 10%	Weak positivity
+2	10–40%	Moderate positivity
+ 3	40% or above of the counted cells	Strong positivity

### Quantitative Analysis for Immunohistochemistry

Images of placenta and POCs were captured using an Olympus BX40 microscope (Artisan Scientific, Champaign, IL, United States) at 10×, 20×, and 40× magnification. The number of cells with positive and negative stain in all the tissue sections was counted by using cell counter function manually.

## Results

### Patient Characteristics

There were 10 cases in the placenta and 10 cases in the POC groups. There were 2 cases in the placenta, 4 in the gestational diabetes, 4 in the pre/eclampsia, and 10 cases in the POC groups. The mean maternal age of patients in Group I was 33.20 ± 2.57 years and that in Group II was 33.20 ± 2.57 years (*P* < 0.05). The mean stain score in each group was 3.0 ± 0.0 (*P* > 0.05; *P* < 0.05) (Table 2).

**TABLE 2:** Patients characteristics.

Characteristics	Placenta (*n* = 10)	POCs (*n* = 10)	*P*-value
No. of cases	10	10	
Maternal age	33.20 ± 2.57	30.40 ± 1.71	0.010**
Stain score	3.0 ± 0.0	3.0 ± 0.0	0.038**

**Independent sample *t*-test.

### α7-nAChR Expression in Normal, PE, and GD Placenta and Product of Conception

**FIGURE 1:**
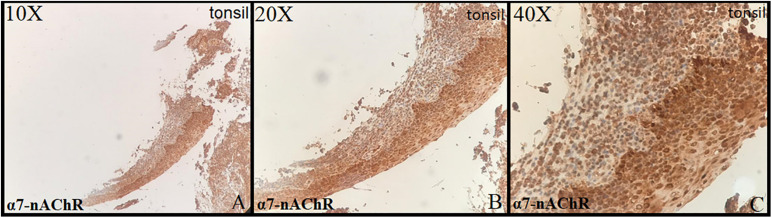
Control: tonsil (10×, 20×, 40×) strong positive stain in the squamous cells of lining epithelium (+3 intensity).

**FIGURE 2:**
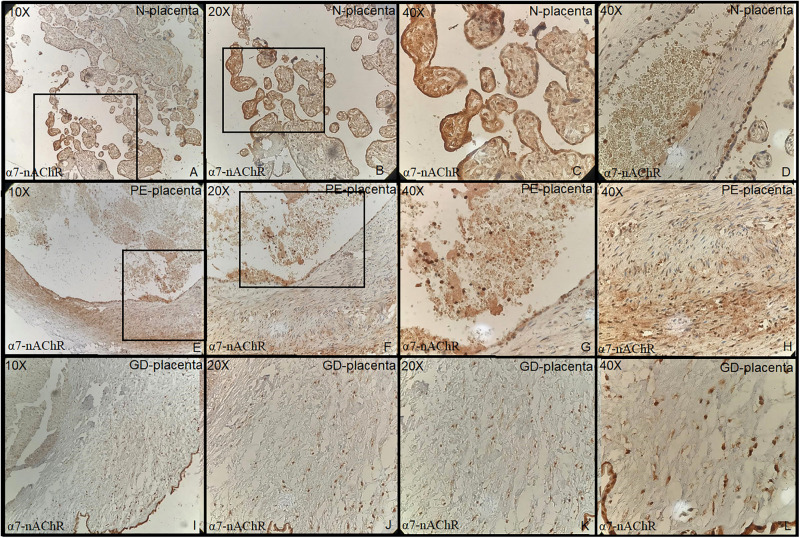
**(A–D)** Normal placenta (10×, 20×, 40×) revealed positive strong staining in chorionic villi lining cells both cytotrophoblast and syncytiotrophoblast. **(E–H)** Preeclampsia: Placenta (10×, 20×, 40×) shows increased thickness of media of the blood vessels walls with positive staining of endothelial cells and myofibroblast. **(I–L)** Gestational diabetes: Placenta revealed strong positivity of endothelial cell and myofibroblast (media is thickened) positive cell line (all + 3 intensity).

**FIGURE 3:**
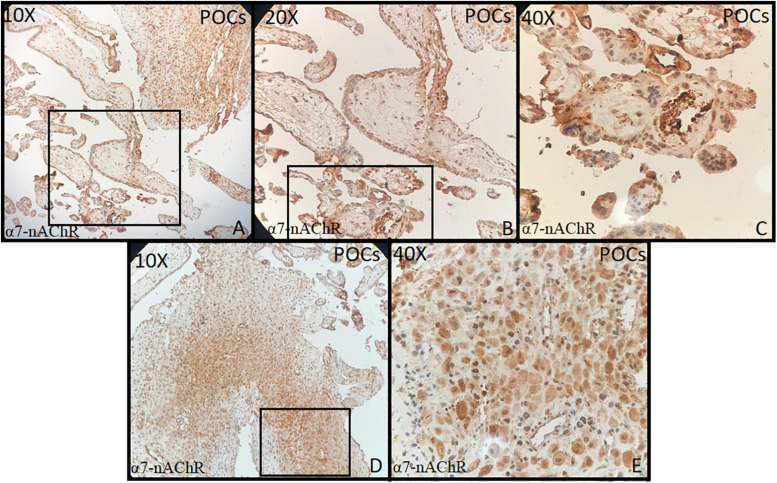
**(A–E)** Products of conception (POCs) (10×, 20×, 40×) showed strong staining in the decidua, endothelial, myocytes of maternal blood vessels, and in syncytiotrophoblast and cytotrophoblast of chronic villi (all + 3 intensity).

Tonsillar tissue was strongly positive with + 3 intensity (Figures 1A–C). Immunohistochemical expression and localization of nAChR were evaluated and observed to be intense in all the cases of PE and GD as well as in the normal placenta (Figure 2) and POCs (Figure 3). α7-nAChR showed no significant differences among different cases of miscarriages irrespective of the mother’s age and gestational age at which the event occurred. The α7 receptor was widely distributed in the umbilical cord, fetal membranes, and placenta. There was intense staining in the decidual cells, trophoblastic cells of fetal membranes, and chorionic villi (Figure 2). The staining was primarily nuclear but also observed in cytoplasmic cells. Within the villi, the expression was highest, with no differentiation among the others (Figure 2). The specific staining pattern of α7-nAChR receptor was observed in the cell nucleus in the same decidua maternal villi in which cytoplasmic staining was well marked in syncytiotrophoblast and cytotrophoblast of chronic villi.

## Discussion

The immunohistochemical expression of α7-nAChR subunit in the three groups of placentas (NP, PE, and GD) as well as in POCs showed villi changes specific to α7. The expression was high in the villi and decidua. To date, few studies have been found regarding the immunohistochemical expression of α7-nAChR in the placenta. This includes Lips et al. (2005) who reported the nAChR messenger RNA (mRNA) expression; Kwon et al. (2007) who reported α7 expression; and a recent study by Aishah et al. (2017, 2019) who reported the mRNA and protein expression of the α3 and β1 subunits. These studies showed that the nAChR subunits were expressed in the placenta, and their levels vary according to the cellular type. Herein, we report that the α7 subunit was more expressed in the villi and decidua and that they did not differ in the intensity of expression.

The study in 2018 by Machaalani et al. (2018) revealed the highest expression of α4 expression in placenta among the nine subunits studied. In our study, the highest α7 was in the normal placental cells of the villi lining cells both cytotrophoblast and syncytiotrophoblast. Increased thickness of media of the blood vessel walls with positive staining of endothelial cells and myofibroblast were seen in the PE and GD groups as compared to the control group. Small sample size is a limitation in our study.

Here, we have reported the protein localization and its expression in terms of intensity of α7-nAChR subunit in placental tissues and POCs in Pakistani population. The other subunits should also be explored in further studies. Additionally, different geographical locations within Pakistan, as well as in other countries, must also be investigated to see any possible differences. Pakistan is an agricultural country, and many banned pesticides are in use, e.g., dichlorodiphenyltrichloroethane (DDT), organochlorines, and organophophates, which have high nicotine content. Furthermore, cigarette smoking and huqqa smoking by men may put women and unborn child at risk due to passive smoking. This situation vary in different countries and may provide a possible link for diagnosis of at-risk individuals, awareness among society, and a direction for policy makers for banning the hazardous pesticides and smoking at public places.

## Conclusion

Immunohistochemical expression and localization was almost similar in all placental groups and POCs. However, some exceptional prominent changes were in the villi in PE and GD groups. There was thickening of the lining of blood vessels in the placenta of these groups, which may be responsible for poor oxygen supply and complications for the fetus. More studies are required to further explore all nicotinic receptors in such cases to find a plausible association with sudden infant deaths, sudden intrauterine deaths, still births, and miscarriages.

## Data Availability Statement

The original contributions presented in the study are included in the article/supplementary material, further inquiries can be directed to the corresponding author.

## Ethics Statement

The studies involving human participants were reviewed and approved by the University of Lahore ethical review board. The patients/participants provided their written informed consent to participate in this study.

## Author Contributions

AA contributed in the planning, write up and designing and facilitated financially and logistically to make this study happen. KE contributed in microscopy and preparing the figures. All the authors have contributed to this study.

## Conflict of Interest

The authors declare that the research was conducted in the absence of any commercial or financial relationships that could be construed as a potential conflict of interest.
